# An Issue on Innovation: What Makes Curricula Innovative and New Approaches to Neurology Education

**DOI:** 10.1212/NE9.0000000000200062

**Published:** 2023-03-10

**Authors:** Roy Strowd

**Affiliations:** From the Department of Neurology, Wake Forest School of Medicine, Winston Salem, NC.

In this first issue of 2023, *Neurology*® *Education* is chock-full of educational scholarship. The issue features the first 2 Reviews in Medical Education articles in the journal. The first article reviews educational innovations in neurology and the second reviews the use of simulation in neurology training. In addition, this issue includes research articles organized in 3 topic areas: enhancing outpatient training in neurology residency, special topics in neurology education, and neurologic education in nonphysician disciplines. Readers, authors, writers, and educators all stand to learn a great deal from this issue.

## Innovations in Neurologic Education

Neurology education is changing. While the coronavirus disease 2019 pandemic catalyzed a wave of innovations in how we teach telemedicine, virtual examination, online learning, distance education, and global neurology, many of these trends began well before the pressures of the pandemic.^1^ In scholarly journals, innovation articles have emerged as a distinct genre of educational scholarship. While different journals have varying criteria for reporting an innovation, common features of a high-quality educational innovation have been defined. A recent scoping review defined 12 aspects of an educational innovation: (1) identifies a problem, (2) presents preliminary work, (3) explains what is novel, (4) presents a conceptual framework, (5) describes the implementation, (6) discusses how it was developed, (7) evaluates the innovation, (8) includes outcome data, (9) reflects on lessons learned, and (10–12) discusses sustainability, transferability, and influence on the field.^2^

How are we doing in reporting high-quality innovations in neurology education? In this issue, a scoping review by Zimmerman et al.^3^ addresses this topic by asking 2 specific scholarly questions: (1) what educational innovations are neurology educators using and (2) how truly innovative are these interventions? The authors reviewed 350 studies and found that the most common educational innovations in neurology were simulation, eLearning, 3-dimensional modeling, virtual reality, and social media studies. The review highlights several strengths of this literature. Of the 12 criteria for reporting a high-quality innovation, 8 of these criteria were included in more than 70% of these articles. Outcome data were common and frequently included Kirkpatrick level 1 outcomes describing learner reactions to the innovation and level 2 outcomes describing gain in knowledge and learning. The review also underscores important areas for improving innovation scholarship. The 3 least common reporting criteria that were included in these articles were explaining what was novel, discussing transferability, and describing sustainability. In addition, Kirkpatrick level 3 outcomes that show behavior change and level 4 outcomes that demonstrate improvement in patient care were rarely reported in papers on simulation and eLearning.

What are readers and authors supposed to learn from this article? The highest quality educational innovations start by describing a clear problem in the education literature. The introduction section should build a logical sequence of arguments that culminate in a clear and concise description of the gap that exists in education literature. This section should clearly discuss what is novel about the innovation. The results section should describe the innovation in enough detail that it can be reproduced including how it was developed, pilot work, the conceptual basis, and how it was implemented. Clear outcome data are needed in the results section. Prior to implementing the intervention, authors should determine how they can measure changes in learner behavior (Kirkpatrick level 3 outcome) or improvements in patient care and system processes (level 4 outcome). Finally, the discussion section needs to present lessons learned and contextualize the transferability and sustainability of the intervention.

## Simulation in Neurologic Education

The second review article summarizes how simulation education has been used in neurology and forecasts innovative ways for simulation to address new challenges in clinical training in the future. Since the launch of *Neurology: Education*, the journal has published several articles on simulation. One of the early article compared standardized patients with manikin models during simulation training for neurology residents. This study demonstrated comparable outcomes—an important finding for educators in resource-limited settings, when standardized patients are unavailable or for topics that do not lend themselves to live patient actors.^4^ Since then, articles have described the successful use of simulation for teaching acute neurologic emergencies and viral encephalitis.^5,6^

Where is simulation training headed? In recent decades, health profession education has witnessed a shift toward competency-based assessment in both undergraduate and graduate medical education. Entrustable professional activities, behavior-informed milestones, and competency-based assessments have been incorporated into expectations for training.^7,8^ However, readily available assessment tools that reliably demonstrate competence and readiness for independent practice have lagged behind these curricular standards. Simulation offers a powerful approach to achieving competency-based practices. Albin et al.^9^ do not stop there and anticipate increasing innovations in how simulation is delivered remotely, at distances, through global partnerships, and with incorporating augmented and virtual reality technology.

What are education scholars and authors supposed to take away from this article? First, there is an opportunity to use simulation to advance diversity, equity, and inclusion (DEI). Albin et al. specifically cite the role of simulation to deliver DEI training for staff and medical trainees. Second, authors reporting the results of simulation studies need to incorporate higher-level outcomes. The impact of simulation curricula on learner behaviors, system-based practices, and patient outcomes are needed. Authors should consider incorporating these outcomes at the outset of their study. Finally, articles reporting simulation interventions need to clearly describe the generalizability and transferability of their approach to resource-limited settings—how could this simulation curriculum be implemented for all, anywhere.

## Outpatient Training, Special Topics, and Advanced Practice Provider Education

The research articles in this issue cover articles on outpatient training, special topics, and training across different disciplines and professions in clinical neurology.

### Enhancing Outpatient Training in Neurology Residency

The 3 articles in this section present important data on why increasing exposure to outpatient neurology is critical now. Doughty et al.^10^ describe the development and implementation of a didactic series where residents learn from patient and physician storytelling. Dyads of patients and their accompanying neurology physician participated in a live interview. Using a conversation style, the pairs discussed patient-centered management of selected neurologic disorders. This curriculum highlighted the power of the patient voice. Residents were provided with a window into what it is like to develop a long-term relationship with your patient and were inspired into careers in the clinic.

### Special Topics in Neurology Education

In this second section of articles, authors' report on approaches to teaching neuro-ophthalmology, comfort with treating patients with intellectual disability, and team-based learning.^11-13^

### Neurologic Education Across Professions and Disciplines

Neurology faces a critical physician shortage. Patient-centered care requires interprofessional collaborative practice where providers across varying disciplines and professions work together to deliver team-based care. Training the next generation of capable practitioners will require evidence-based educational approaches to teaching clinical neurology to all disciplines and professions. We need to understand where the gaps are in teaching advanced practice providers (APPs), nurses, therapists, pharmacists, technologists, and others in the care team. We need innovation in training within and across these programs. This is a priority area for *Neurology: Education*. The journal aims to publish educational scholarship in all these disciplines, professions, and neurology adjacent fields. The 2 articles in this section address gaps in training for APPs and internists. Harrison et al.^14^ identify important gaps in the training of APPs that hinders exposure to neurology and may reduce the likelihood of APPs pursuing neurology-related fields following graduation. Sanky et al.^15^ deconstruct the consult process and emphasize the often-discordant expectations between neurology and internal medicine residents. This article reminds educators of the importance of imbedding trainees in areas outside of their discipline and profession to align expectations, create collegiality, and encourage collaboration.

With 10 articles in this issue, there is plenty of great educational scholarship to consume. Enjoy this issue of *Neurology: Education*. Happy reading.
